# Abundance and Diversity of *Culicoides* Species (Diptera: Ceratopogonidae) in Different Forest Landscapes of Karnataka, India: Implications for *Culicoides* Borne Diseases

**DOI:** 10.1155/2023/6250963

**Published:** 2023-08-31

**Authors:** Munivenkatarayappa Archana, Nayankumar, Rajamanikandan Sundarraj, Arpita Giddobanahalli Mruthyunjaya, Taniya Ghosal, Abhijit Mazumdar, Divakar Hemadri, P. P. Sengupta, Minakshi Prasad, Yella Narasimha Reddy, Krishnamohan Reddy Yarabolu, Janofer Ummer, Jyoti Misri, Habibar Rahman, Bibek Ranjan Shome, Sathish Bhadravati Shivachandra, Mohammed Mudassar Chanda

**Affiliations:** ^1^Department of Animal Husbandry and Veterinary Services, Government of Karnataka, Bengaluru, India; ^2^Centre for Drug Discovery, Department of Biochemistry, Karpagam Academy of Higher Education, 641021, Coimbatore, Tamil Nadu, India; ^3^IQVIA, Bangalore 560103, India; ^4^The University Burdwan, Burdwan, West Bengal, India; ^5^ICAR-National Institute of Veterinary Epidemiology and Disease Informatics (NIVEDI), Bengaluru, Karnataka, India; ^6^Department of Animal Biotechnology, Lala Lajpat Rai University of Veterinary and Animal Sciences and National Research Centre on Equines, Sirsa Road, Hisar 125001, Haryana, India; ^7^Department of Animal Biotechnology, P.V. Narsimha Rao Telangana University, Hyderabad, Telangana, India; ^8^Tamil Nadu University of Veterinary and Animal Sciences (TANUVAS), Chennai, Tamil Nadu, India; ^9^Indian Council of Agricultural Research (ICAR), Krishi Bhawan, New Delhi, India; ^10^International Livestock Research Institute, NASC complex, Pusa, New Delhi 110012, India

## Abstract

*Culicoides* are important vectors for livestock and human pathogens. Wild animals act as reservoirs for important orbiviruses such as bluetongue and African horse sickness viruses. There are only limited studies on the distribution of *Culicoides* species in forest habitats. In this study, we collected *Culicoides* from different wildlife sanctuaries and national parks of Karnataka. We collected and morphologically identified 8597 *Culicoides*. We found 18 species of *Culicoides* in different sites, with *C. oxystoma* and *C. imicola* being the predominant species across the sites. The sequence alignment and phylogenetic analysis of the Cox1 gene of *Cuilicoides* species revealed a huge level of sequence similarity and their wide distribution around the world. Most of the isolates from our study were closely related to Chinese isolates. The abundance of the species was analyzed using the Bayesian ordination method. We used a hierarchical joint distribution negative binomial regression model to detect the correlation between species owing to environmental covariates and residual correlation. The presence of potential vectors for important livestock pathogens in wild habitats in our study warrants further research on the detection of pathogens in *Culicoides* collected from forest habitats and adopt surveillance in wild animal habitats to prevent disease spread from wild animals to livestock and vice versa.

## 1. Introduction

*Culicoides* biting midges (Diptera: Ceratopogonidae) can transmit economically important diseases such as bluetongue virus (BTV), African horse sickness virus (AHSV), epizootic hemorrhagic disease virus (EHDV), Schmallenberg virus (SBV) in animals and can also transmit pathogens like Oropuche virus in humans [1]. India has had regular outbreaks of BTV since it was detected first time in 1963 [2], resulting in huge economic losses for subsistence farmers [3]. Bluetongue has emerged in new areas, such as Northern Europe, and has caused huge losses in the agricultural sector. Therefore, the epidemiology of bluetongue and its vectors needs to be studied [4].

In addition to domestic livestock species, wild ruminants can also be affected by *Culicoides*-borne diseases such as bluetongue [5]. Wild ruminants have been linked to bluetongue outbreaks [6]. Outbreaks of BTV and EHDV in cervids, especially white-tailed deer, have also been reported [7]. Bluetongue and EHDV are very common in domestic animals in North America, and a surveillance system is in place to monitor the presence of these viruses in wild ruminants and in vectors [8]. In Europe, there are many studies to understand the role of wild ruminants in the transmission of BTV [9, 10]. There is an apparent increase in the occurrence of hemorrhagic diseases in the United States [11]. The occurrence of epizootic hemorrhagic disease in white-tailed deer is determined by the subspecies due to the influence of innate disease resistance [12]. However, such studies are lacking in Asian countries, including India.

There are more than 1,000 species of *Culicoides* worldwide [13], 27 of which have been reported from India [14, 15]. An abundance of vectors and the differential feeding preferences of vectors can determine the risk of *Culicoides*-borne diseases [16]. *Culicoides* feed on many vertebrates and can be opportunistic feeders [17]. The majority (65%) of blood meal sources studied are from vertebrates, with cattle as the preferred host for many *Culicoides* species [18]. In the past, outbreaks of bluetongue in Karnataka, Tamil Nadu, and Andhra Pradesh have been associated with periods of peak *Culicoides* activity [19, 20]. *Culicoides actoni*, *C. anophelis*, *C. inoxius*, *C. majorinus*, *C. peregrinus*, *and C. oxystoma* were reported from Chittor and Prakasam districts of Andhra Pradesh [21]. The reasons for this diversity in the distribution of *Culicoides* species and the presence of different serotypes are not known. DNA barcoding of *Culicoides* species was performed for 12 species from Tamil Nadu [22], and it was found that for most species from other countries, matching sequences are available in the GenBank. Systematic surveillance of *Culicoides* is lacking in India. Systematic studies are needed in different parts of India to establish the exact number of *Culicoides* species in India [19] and also their role in the transmission of bluetongue and other orbiviruses. In addition, there are very few studies on vector surveillance or systematic studies of vector distribution in forest landscapes in other countries [7, 23, 24] and none in India. The BTV segment was detected in *Culicoides pulicaris* caught near recent BT outbreaks in Belgium using real-time reverse transcription polymerase chain reaction (PCR) [25].

Most work on modeling of *Culicoides* species considers individual species (as a response variable) in order to understand the spatial distribution and seasonality [26, 27]. However, in ecology, the inference should be drawn at the community level rather than at the individual species level in order to better understand the density-dependent and density-independent factors responsible for species abundance. For example, we might be interested in how the different *Culicoides* species vary across sites and how these differences are influenced by either environmental covariates or are due to interactions between the species. Multivariate abundance data are most often analyzed using data ordination methods such as Non-metric multidimensional scaling (NMDS) [28], principal coordinate analysis [29], and detrended correspondence analysis [30]. These methods process the data in a series of steps to extract meaningful information. However, these methods do not assume an underlying distribution or a statistical model and, therefore, can be categorized as nonmodel-based methods [31]. The other approach to analyze multivariate abundance data is using model-based approaches [32]. Compared to the nonmodel-based ordination methods, model-based methods are more advantageous for community-level inference [31]. The hierarchical Bayesian modeling approach is gaining importance in the analysis of multivariate abundance data [33–35].

Characterization of *Culicoides* communities in areas where wild ruminants occur is lacking in India. The main aim of this study was to collect and identify *Culicoides* species from different forest landscapes and how the abundance of these species is influenced by climate and geographical location. We used Bayesian Analysis of Multivariate Abundance Data in Ecology (BORAL) to analyze the abundance of *Culicoides* species at different sites in our study [36]. In BORAL, many models can be fitted with Bayesian Markov Chain Monte Carlo estimation using covariates, or we can include latent variables to model the correlation between species. The advantage of using latent variables is to account for residual correlation between species due to biotic interactions or missing covariates.

The present study was designed to target different forest landscapes and at the wildlife-livestock interface to understand the species similarity and diversity across the sites. Karnataka state was selected for this study, as it is one of the endemic southern states for bluetongue [3].

## 2. Materials and Methods

### 2.1. Selection of Sites for Trapping

#### 2.1.1. Traps, Collections, and Separation of *Culicoides*

*Culicoides* were collected by using light traps (UV– LED–CDC light traps) from different forest habitats based on the forest classification and also at the wildlife-livestock interface (Table 1). The wildlife sanctuaries (WLS) and national parks (NP) of Karnataka were selected for trapping, and permission was obtained from the Principal Chief Conservator of Forest (PCCF-Wild Life), Karnataka to set up insect light traps. Three WLS were selected for trap placement. A total of 18 sites were selected based on *Culicoides* habitat and also wild ruminants sightings within three Karnataka WLS and NPs (Table 1). Global Positioning System (GPS) measurements (latitude and longitude) were recorded for all locations. A format for location information and species identification was created to store and compile the data. A database was developed to record all species and age gradation data. The domestic sites were also selected based on the coverage of the forest class. Out of 18 sites, 6 sites were domestic sites (Table 1). Seven sites were also chosen at the wildlife-domestic interface to compare the collections and the possibility of a spillover of the disease from wild to domestic animals and vice-versa. Collections were made during dawn and dusk, and traps were set from 6:00 pm to 6:00 am once per month for 8 months for all sites, except for one site wherein five collections were made (Table 1). The light traps were placed in locations such as near damp habitats and near water bodies and were hung at night on the walls of buildings/trees at 1.5–2.0 m from the ground in forest habitats. The collected insects were transported to the laboratory in the collecting beakers filled with water and then recovered and preserved in 70% ethanol for further investigation.

### 2.2. Morphological Identification

First, the insects were sorted into *Culicoides* and non-*Culicoides* for further identification. The sorted specimens were separated into different species using morphological methods. Each species was first separated into males and females. The female specimens were age-graded (nulliparous, parous, gravid, and blood engorged). *Culicoides* were identified morphologically at the species level using published keys [37–41]. Female specimens were further classified as unpigmented, pigmented, gravid, blood-fed, and stored in 70% ethanol (with the exception of blood-engorged specimens, which were stored in 90% ethanol) and stored at −20°C until further molecular identification. Pictures of full body *Culicoides* were taken in a stereo zoom microscope (Zeiss^R^) under 5x magnification.

#### 2.2.1. Nondestructive Method of DNA Extraction from *Culicoides* and Molecular Identification of *Culicoides*

Total DNA was extracted from individual *Culicoides* specimens using a nondestructive technique as described [22]. Amplification of DNA barcode segment of mitochondrially encoded cytochrome oxidase subunit I (COI) region primers LCO1490 (5′-GGT CAA CAA ATC ATA AAG ATA TTG G-3′) and HCO2198 5′-TAA ACT TCA GGG TGA CCA AAA AAT CA-3′) [42] were used for molecular identification of *Culicoides* species. Amplification of a 658 bp fragment of the mitochondrial COI gene barcoding region was achieved by PCR. Reactions were performed in a total volume of 25 *μ*l, consisting of 5 *μ*l of nuclease-free water (Genetix), 12.5 *μ*l of 2x Master Mix (Genetix, India), 1.25 *μ*l of the 20 *μ*M forward primer LCO1490, 1.25 *μ*l of the 20 *μ*M reverse primer HCO2198 and 5.0 *μ*l of template DNA for each reaction. Positive and negative controls for the amplification reactions were performed on each round of PCR. The PCR cycling conditions were as follows: an initial denaturation step at 94°C for 3 min followed by 35 cycles at 94°C for 30 s, 46°C for 30 s, 72°C for 1 min, and a final extension step at 72°C for 10 min. The reactions were stored at 4°C until further processing. After completion of the PCR reaction, 5 *µ*l of amplified products were electrophoresed in 1.5% agarose gel together with 6x gel loading dye, and a 100 bp DNA ladder was used as a marker. The images were captured using gel documentation system. The resulting PCR products were sent for bi-directional sequencing using primers LCO1490 (forward) and HCO2198 (reverse) (Eurofins, Bengaluru, India). The resulting electropherograms were edited, and forward and reverse sequences were reassembled and trimmed to remove the primer sequence using Bioedit software. Corresponding specimen collection data and DNA sequences, including electropherograms, have been submitted to the GenBank database. The voucher specimens were prepared for the samples used for molecular identification for comparison of morphological and genetic data. Wing pictures of the voucher specimens (after nondestructive DNA extraction) were taken in a stereo zoom microscope under 5x magnification (Zeiss^R^).

#### 2.2.2. Phylogenetic Analysis

In order to understand the evolutionary relationship of the Cox1 gene cytochrome *c* oxidase subunit isolated from the *Culicoides* species in the present study along with the Cox1 gene isolated from other parts of the world, the evolutionary analysis was carried out. The 25 Cox1 genes of *Culicoides* species were isolated in this study and were further sequenced and deposited in the NCBI database. Apart from our isolates, all the Cox1 genes from *Culicoides* species available in the NCBI database were retrieved in FASTA format for evolutionary significance. To reduce false negatives, features like pseudo genes, partial sequences, and repeated sequences were manually removed. ClustalW [43], an algorithm implemented in the Mega 11 (Molecular Evolutionary Genetics Analysis) program [44], was used to perform multiple sequence alignment. The phylogenetic tree was constructed from the aligned sequences using the Mega 11 default parameters; the neighbor-joining method (Maximum Composite Likelihood matrix) was used to infer the evolutionary history between the sequences [45, 46]. The phylogenetic tree's robustness was evaluated using 100 bootstrap replication steps [47]. We have constructed two individual phylogenetic trees to decipher the significance with the species and among the species of *Culicoides*.

### 2.3. Analysis—Heat Maps, BORAL Models

We used aggregated (sum over eight months period) *Culicoides* species data (number of species = 18) from 15 different sites. A total of 12 temporally Fourier processed remotely sensed variables (minimum, maximum, and variance of day and night land surface temperature, enhanced vegetation index, normalized vegetation index) [48] were extracted for the sites and used as environmental variables for the analysis.

We fitted five different models to our data; purely latent model, Poisson model with and without covariates, and negative binomial model with and without covariates. Model description is given below:

1. Purely latent model

(1) $$\begin{array}{c} \log\left(\mu_{ij}\right)=\alpha_{i}+\theta_{0j}+z_{i1}+\theta_{j1}+z_{i2}X\theta_{j2}=\alpha_{i}+\theta_{0j}+z_{i}T\theta_{j}, \end{array}$$

where *μ*_*ij*_ is the mean response at site *I* for *Culicoides* species *j*, *θ*_0*j*_ is the species-specific intercept, *z*_*i*_=(*z*_*i*1_, *z*_*i*2_)^*T*^ is a vector of two latent variables, and *θ*_*j*_=(*θ*_*j*1_, *θ*_*j*2_)^*T*^ are the corresponding species-specific coefficients.

(2) Correlated response. We used different remotely sensed variables to explain the correlation between different *Culicoides* species.

(2) $$\begin{array}{c} \log\left(\mu_{ij}\right)=\theta_{0j}+NDVI_{i}X\beta_{j1}+dLST_{i}X\beta_{j2}+nLST_{i}X\beta_{j3}+,\ldots ,+\theta_{j1}+z_{i}T\theta_{j}=\theta_{0j}+x_{i}{\;}^{T}\beta_{J}+z_{i}T\theta_{j}, \end{array}$$

where *μ*_*ij*_ is the mean response at site *I* for *Culicoides* species *j*, *θ*_0*j*_ is the species-specific intercept, *z*_*i*_=(*z*_*i*1_, *z*_*i*2_)^*T*^ is a vector of two latent variables, and *θ*_*j*_=(*θ*_*j*1_, *θ*_*j*2_)^*T*^ are the corresponding species-specific coefficients.

Where *x*_*i*_ is a vector with 12 covariates and their co-efficients *β*_*j*_=(*β*_*j*1_,…,*β*_*j*12_)^*T*^.

### 2.4. Model Checking

The residual analysis of various models was checked with Dunn–Smyth residuals [49]. The residual versus predicted response plots were used to check for overdispersion and potential outliers. Quantile plots of the residuals were used to assess the goodness of the fit.

### 2.5. Model Selection

We have built in four models; the Poisson model with and without covariates, the negative binomial with and without covariates; the best model was selected based on the lowest deviance information criteria (DIC) [50]. Caterpillar plots were made with 95% highest posterior density intervals (HPD) from the best model with covariates to know the significant effect of environmental (remotely sensed variables) variables on different species.

## 3. Results

**Figure 1:**
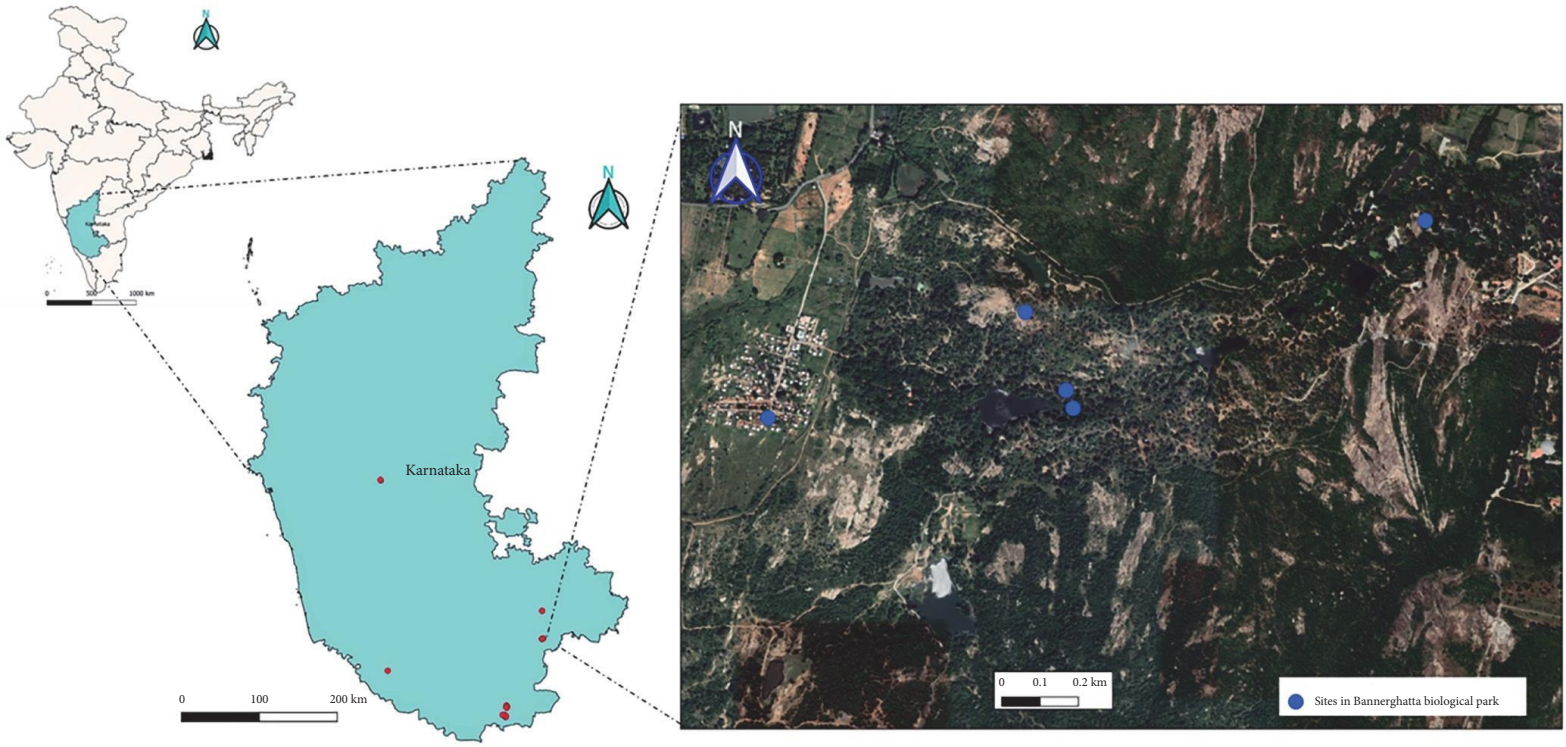
Sites within Bannerghatta Biological Park.

**Figure 2:**
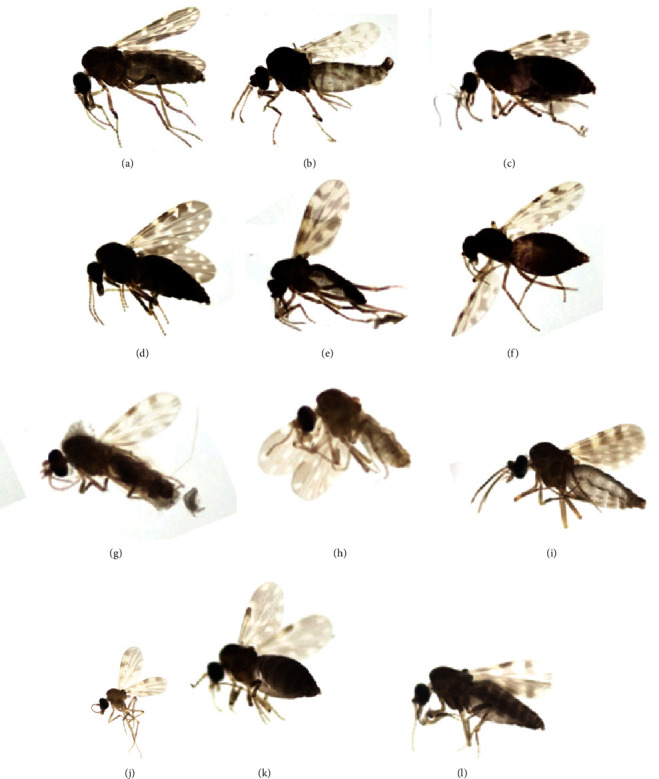
Images of different *Culicoides* species captured in stereo zoom microscope. (a) *C. arakawae*, (b) *C. circumscriptus*, (c) *C. oxystoma*, (d) *C. peregrinus*, (e) *C*. *orientalis*, (f) *C*. *imicola*, (g) *C*. *fulvus*, (h) *C*. *huffi*, (i) *C*. *innoxius*, (j) *C*. *anophelis*, (k) *C*. *similis*, (l) *C*. *brevitarsis*.

A total of 30 traps were placed across all locations. A map showing locations sampled in Bannerghatta national park is shown in Figure 1. The number of collections at each location varied, with a maximum of collection for 8 months and a minimum of five monthly collections due to logistic reasons. Details of the sites with the number of months for which collections were made are given in Table 1. Overall, there were 30 collection sites, and 129 collections were made from different locations at different time points starting from August 2016 until February 2017 (Table 1). A total of 8,597 specimens of *Culicoides* were collected from different sites and collections. Pictures of different *Culicoides* species are shown in Figure 2, and the wing pattern of voucher specimens obtained after nondestructive DNA extraction is shown in Figure S7. Representative samples (in duplicate) from each site for each species were taken for bi-directional sequencing. More than 200 *Culicoides* samples were sequenced. Phylogenetic analysis of 25 sequences for representative species is presented.

### 3.1. Phylogenetic Analysis

**Figure 3:**
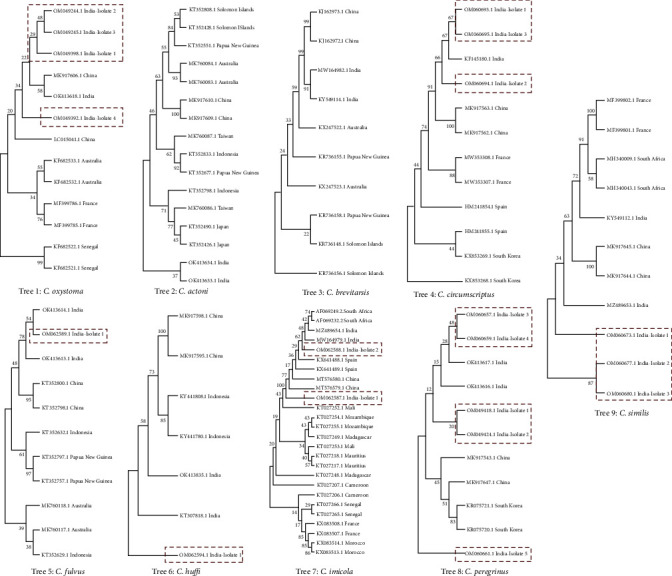
Species-level phylogenetics analysis of Culicoides (within species). The sequences isolated and deposited in NCBI from this study are highlighted in red color rectangular boxes.

The nucleotide sequence alignment of the Cox-1 gene showed 584 parsimony sites, 15 conserved regions, 606 variable sites, and 21 singleton sites with an overall average mean distance of 0.73. The length of the Cox1 gene varied in each species, in which the smallest length of Cox1 (337 bp) was reported from *C. orentialis* (Australia isolate) in GenBank from other studies, and it may be due to the incomplete sequencing information and the longest of 707 bp was observed in *C. arakawae* (China isolate), and the average length among the species was 589 bp. Based on the individual species level phylogenetic analysis, we observed that our isolates of the Cox1 gene from *C. oxystoma*, *C. arakawae*, *C. brevitarsis*, and *C. similis* (Tree 1, 4, 5, and 12) were closely related to the existing Chinese and Indian isolates available in GenBank (Figure 3). In addition, *C. huffi* isolates revealed a high percentage of similarity with existing India isolates (Tree 6). *C. imicola* isolates showed high within-species variability, in which our Indian isolates are grouped together with South Africa, Spain, and Chinese isolates (Tree 7). All our Indian *C. peregrinus* isolates were grouped in a single clade and are closely related to Chinese isolates (Tree 8).

**Figure 4:**
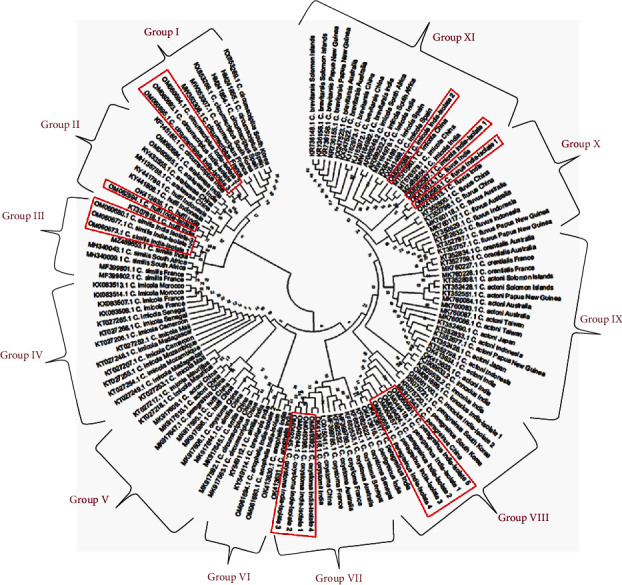
Distribution of Cox1 genes in *Culicoides* genus isolated from various parts of the world (among species).

The overall phylogenetic analysis among the 12 species of *Culicoides* was constructed using neighbor-joining by employing the maximum composite likelihood approach showed that there are 11 different groups (Figure 4). *C. circumscriptus* species from South Korea, France, Spain, and India are branched in Group I. *C. arakawae* (India, China, and Indonesia) and *C. huffi* (Indonesia and India) are categorized in Group II. Group III showed a similar type of branching architecture as of Group II with *C. similis* (India, South Africa, and France). Group IV showed numerous branches, and each entity corresponds with *C. imicola* from Morocco, France, Senegal, Camersoon, Mali, Madagascar, Mozambique, and Mauritius. Six different species, including *C. oxystoma*, *C. peregrinus*, *C. huffi*, *C. oxystoma*, *C. similis*, and *C. circumscriptus* from Chinese isolates are grouped in Group V. Indian isolates of *C. similis*, *C. anopheles*, and *C. brevitarsis* are grouped in Group VI. *C. oxystoma* isolates from five different countries such as India, China, France, Australia, and Senegal, categorized under Group VII. *C. peregrinus* species from India, China, South Korea, and four different *C. innoxius* isolate from India is placed in Group VIII. Group IX contains *C. actoni* from various countries, including India, Indonesia, Japan, Papua New Guinea, Taiwan, Australia, and Solomon Island. Apart, *C. orentialis* from France and Australia were also categorized in Group IX. Single *C. fulvus* species from Papua New Guinea, Australia, China, and India are placed in Group X, and *C. imicola* from various countries, including China, India, Spain, South Africa, Australia, Papua New Guinea, and Solomon Island, are in Group XI.

**Figure 5:**
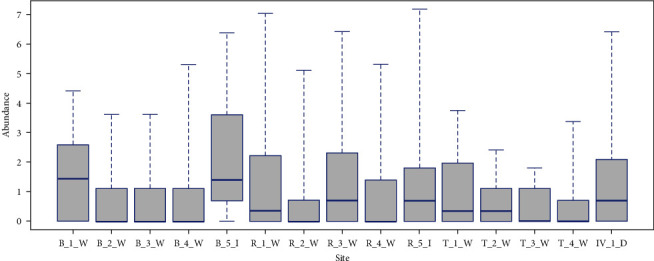
Box-plot showing site-wise variation in abundance of total Culicoides (B_1_W, B_2_W, B_3_W, B_4_W, B_5_I: sites withing Bannerghatta biological park; R_1_W, R_2_W, R_2_W, R_4_W, R_5_I: sites within Ranebennur Black Buck Sanctuary; T_1_W, T_2_W, T_3_W, T_4_W: sites within Biligiri Rangaswamy Temple Tiger Reserve; IV_1_D: Ramagondanahalli Site).

**Figure 6:**
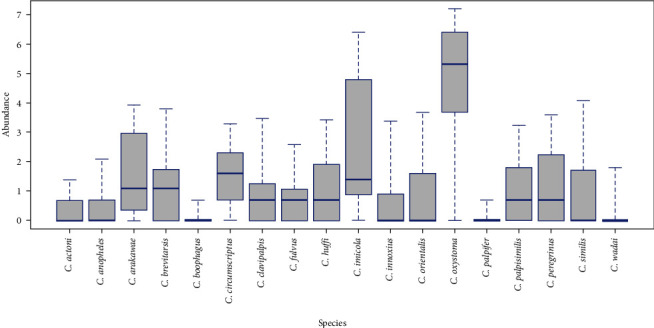
Box-plot showing site-wise variation in species-wise abundance across all sites.

The boxplot showing the site-related variation in the abundance of total *Culicoides* shows that there is variation in the total abundance between sites (Figure 5). Box plot showing the variation in abundance of different *Culicoides* species at each site (Figure 6). Different species occurring at different sites show that the maximum number of species (*n* = 14) at one site in Bannerghatta National Park (B_3_W) and the lowest number of species (*n* = 6) at BRT sites (T_3_W and T_4_W) (Table 2). The diversity indices show that the sites within Bannerghatta National Park and BRT have high scores compared to other sites (Table 3).

**Figure 7:**
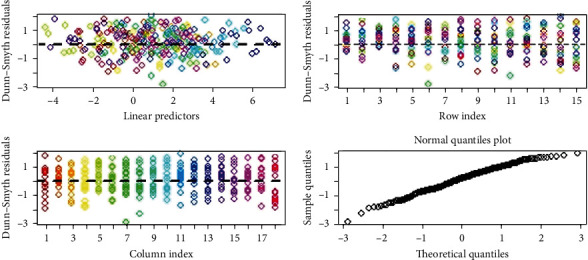
Plots of residual analysis of the negative binomial model without covariates top left: Dunn–Smyth residuals vs. linear predictors, top right: Dunn–Smyth residuals vs. row index; bottom left: Dunn–Smyth residuals vs. column index; bottom right: normal quantile plot of Dunn–Smyth residuals. Funneling effect is observed in the top left plot indicating overdispersion. There is no funneling effect seen here, indicating negative binomial model is appropriate for this data.

The plots of the residual analysis of the negative binomial model with no covariates (Figure 7) show that the negative binomial model is appropriate for the present data. Similarly, a comparison of the models shows that the model with negative binomial distribution and covariates is the best model with the lowest DIC (DIC = 892) (Table 4).

**Figure 8:**
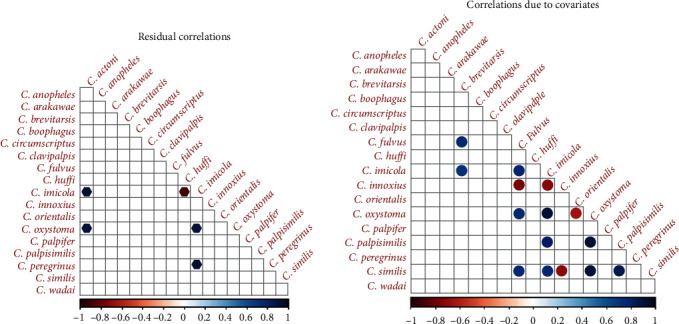
Plot of correlations between species using negative binomial model due to effect of covariates (a) and residual correlations based on correlated response model (b). Significant correlation based on the 95% credible intervals excluding zero is plotted. Red indicates negative correlation, and blue indicates positive correlation. Size of the circle increases with an increase in the strength of correlation.

The correlation between species due to environmental covariates and the residual correlation after accounting for the influence of covariates is shown. There is a correlation between *Culicoides* species due to covariates. There was a significant positive correlation between *C. fulvus* and *C. brevitarsis*, *C. imicola*, *C. innoxius*, *C. oxystoma* (*r*^2^ = 0.75, *r*^2^ = 0.78, *r*^2^ = 0.77, *r*^2^ = 0.76). There was a significant positive correlation between *C. oxystoma* and *C. imicola*, *C. palpisimilis*, *C. similis* (*r*^2^ = 0.92, *r*^2^ = 0.91, *r*^2^ = 0.90) and a negative correlation with *C. orientalis* (*r*^2^ = −0.64). There was a significant positive correlation between *C. palpisimilis* and*C. imicola*, *C. similis* (*r*^2^ = 0.82, *r*^2^ = 0.85). There was a significant positive correlation between *C. similis* and *C. fulvus*, *C. imicola*, (*r*^2^ = 0.77, *r*^2^ = 0.76) and a negative correlation with *C. innoxius* (*r*^2^ = −0.70). There was a significant negative correlation between *C. imicola* and *C. innoxius* (*r*^2^ = −0.73) (Figure 8).

**Figure 9:**
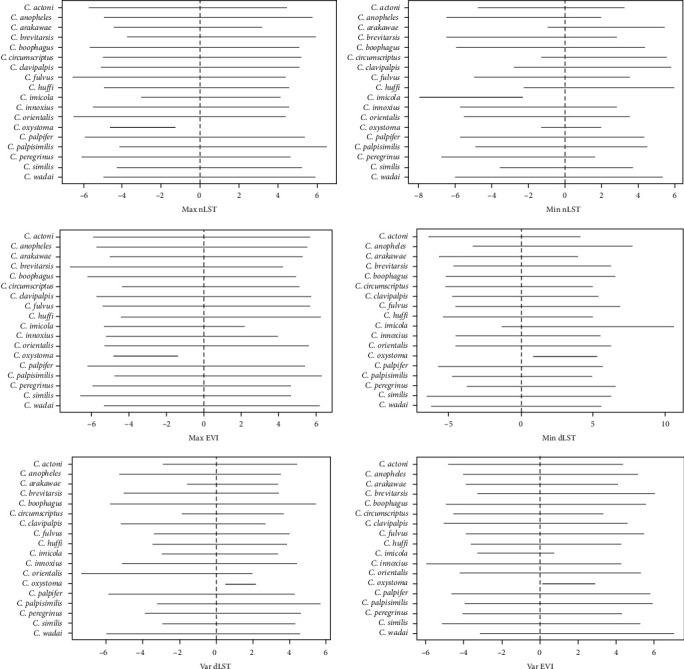
Caterpillar plot of different environmental variables on different species. Horizontal line plots are point median estimates and 95% highest posterior density. Vertical dotted line is to denote the zero values. The HPD values that include zero are shown in gray color, and HPD intervals that do not include zero are shown in black color (significant).

The was also a residual correlation after accounting for the influence of environmental covariates. There was a significant positive correlation between *C. imicola* and *C. actoni*, *C. oxystoma*, *C. peregrinus* (*r*^2^ = 0.92, *r*^2^ = 0.90, *r*^2^ = 0.92) and a negative correlation with *C. huffi* (*r*^2^ = −0.91). There was a significant positive correlation between *C. oxystoma* and *C. actoni* (*r*^2^ = 0.90) (Figure 8). A caterpillar plot of different environmental variables on different species is shown in Figure 9. There is a significant effect of maximum nighttime LST (negative), minimum (positive), variance (positive) daytime LST, maximum (negative), and variance (positive) of EVI on *C. oxystoma* and minimum nighttime LST on *C. imicola* (negative).

## 4. Discussion

Monitoring of *Culicoides* in forest landscapes is important considering the emergence of new diseases such as Schmallenberg and to understand the role of reservoir hosts in the epidemiology of existing diseases such as BTV, EHDV, and AHSV. In general, it is very difficult to capture wild animals and collect samples to detect the presence of viruses and/or antibodies. Therefore, the collection of *Culicoides* species in forest landscapes with a population of wild animals can be an alternative noninvasive surveillance technique.

The present study is the first of its kind in India to investigate the diversity of *Culicoides* species found in forest landscapes of the region. Eighteen species of *Culicoides* have been identified, including potential vectors for bluetongue and other orbiviruses. The species were confirmed by molecular methods. There were significant differences in species diversity between sites. Further, the model-based analysis identified correlations between species that may aid in the future development of vector control strategies. Future studies are needed to identify the microbiome of different species of *Culicoides*.

The presence of 18 different *Culicoides* species identified in our study was similar to other studies in two zoos of South Carolina [24]. Other studies identified more species, such as 20 species in UK zoos [23], 37 species in the National Zoological Gardens of South Africa [51], and 25 *Culicoides* species recorded at a zoo in England [52]. The variation in species numbers in zoos and national parks in other countries can be attributed to the influence of the climate and host abundance. In our study, most of the sites were entirely forest landscapes with the presence of hosts spread across the landscape and not confined as in zoos. *Culicoides oxystoma* was found at all the sites except one site in BRT (T_4_W). The differences in species composition and abundance in different forest types suggest that certain forest types favor the breeding of certain *Culicoides* species. The variation in species diversity may also be due to the diversity of wildlife in the WLPs and NPs in our study. Wildlife data for each site was not available and was therefore not included in the analysis.

The presence of more than 18 species in the forest landscape is significant given the risk of *Culicoides*-borne diseases in wildlife and the possibility of transmission to domestic animals. An outbreak of AHSV occurred in Spain due to the import of zebras from Namibia [53], and more recently, an outbreak occurred in Thailand, representing a significant geographical spread of the virus [54]. Therefore, guidelines are needed for the importation of reservoir animals such as zebra into India, which can pose a serious threat to equine health in India. The presence of potential vectors for AHSV and a suitable environment always poses a risk for AHSV and other midge-borne equine diseases [55].

The sequence alignment and phylogenetic analysis of the Cox1 gene of *Culicoides* species revealed huge levels of sequence similarity and their wide distribution among various countries. In addition, many of the Indian isolates were observed to be closely related to Chinese isolates.

*C. imicola* and *C. oxystoma*, the main species found in India, are found in distinct clusters, which is interesting given the seasonality of these species. All other species are in one cluster (Figure S4), which is an interesting finding, and further studies are needed to map their breeding habitats so that targeted vector control measures can be considered. The clustering of the sites shows that site 3 within the Bannerghatta National Park is in one cluster. This site is near a lake, and 14 species of *Culicoides* have been found at this site. The other interesting result of the hierarchical cluster analysis, is that a village with a large livestock population, adjacent to Bannerghatta National Park (B_5_I), and the cattle shed in the Ramagondanahalli village (IV_1_D) are in a cluster, indicating the influence of domestic livestock population on abundance of *Culicoides* species (Figure S5).

*Culicoides* species feed on a wide range of mammals and birds, but not all are potential vectors of BTV or SBV. Therefore, other species can serve as potential bridge vectors by circulating the pathogens between wild and domestic ruminants [56]. The majority of *Culicoides* species feed on a wide variety of mammalian species [57]. *Culicoides* species can bite a wide range of vertebrate species, with mammals and birds being preferred [58]. However, *Culicoides* species are opportunistic for their blood meal host but prefer cattle when they are present [59]. There have been reports of more bluetongue cases in domestic ruminants where wild ruminants, particularly red deer, are present, and therefore, further research is needed at the wildlife-host-vector-pathogen interface [60]. BTV infections have been reported in African carnivores, and it is hypothesized that the infection is due to the ingestion of meat from animals infected with BTV [61]. However, there is also evidence for the role of *Culicoides* in the transmission of BTV to domestic dogs fed commercially available canned food [62]. This shows the wide range of hosts that can be bitten by *Culicoides* species, including carnivores, indicating the potential role of other hosts in the transmission of bluetongue and other orbiviruses. While *C. imicola* has been implicated as a potential vector for bluetongue and AHSV, the importance of local species cannot be ignored. The first appearance of the novel Schmallenberg virus in 2011 with strong involvement of the Obsoletus species complex (*C. obsoletus and C. scoticus*), but less prevalence in *C. imicola*, underscores the importance of local species in the emergence of new viruses [63]. There have been reports of BTV circulating in wild ruminants even after vaccination in domestic animals, suggesting the need for better surveillance and control measures in wild animals [64]. There is evidence of the presence of BTV/EHDV antibodies in free-ranging black bears [65]. We have recorded potential vectors (e.g., *C. imicola*) in domestic sites, forest sites, and at the wildlife-livestock interface, suggesting the possibility of disease spread, as the dispersal of *Culicoides* species has been observed up to 3.125 m [66].

We have recorded both mammalophilic and ornithophilic species in our collections. The presence of sensory structures is used to distinguish ornithophilic and mammolophilic species [67]. We have detected *C. circumscriptus*, which can transmit hemoproteus parasites, at 12 of the 15 sites, indicating its wide distribution for potential disease spread [68].

In our modeling results, we found that the negative binomial model with covariates outperformed the Poisson model with and without covariates. The influence of environmental covariates on the correlation between *Culicoides* species shows that there is a significant positive and negative correlation between species, and this could be identified by using the Bayesian joint distribution model in our study. The notable finding is a positive correlation between *C. oxystoma* and *C. imicola* and a negative correlation between *C. orientalis* and *C. oxystoma*. *C. oxystoma*, and *C. imicola* have been reported together from many studies in India [69–71], and similar environmental conditions may favor their breeding, but the seasonality of these two species may vary as indicated in our monthly data (data not shown). Interestingly, *C. orientalis* and *C. oxystoma* are negatively correlated, suggesting that different environmental conditions favoring their abundance, but further studies are needed to understand the habitat preferences of these two species as *C. orientalis* is considered to be a bridge vector [72]. The correlation due to the influence of environmental variables can also be attributed to density-independent factors influencing species abundance. The residual correlation after accounting for the influence of environmental variables also shows a positive correlation between *C. imicola* and *C. oxystoma*. Residual correlations, after accounting for the influence of environmental covariates, can be attributed to a density-dependent phenomenon. Other studies use separate models to identify the effects of environmental variables on species abundance, rather than using joint distribution models as in this study, using all *Culicoides* species as response variables. Separate logistic regression models for *C. imicola* and *C. obsoletus* and areas of spatial coincidence of the two vectors were identified [73]. Species-specific models have also been developed using temporally Fourier-processed remote-sensed variables. It was found that the inclusion of non-climatic environmental variables may improve modeling results [74]. This shows the importance of accounting for missing variables with latent variables, as was done in our study using latent variables. The nonparametric Mann–Whitney test was used to test the significance of environmental, topographical, and climatic factors for *Culicoides* species groups [75]. Independent assessment of the correlation between species groups and the influence of environmental variables cannot account for the inherent species interactions and can introduce bias in the estimates. A hierarchical generalized linear model framework was used independently to study the influence of landscape, host, and remotely sensed variables on the abundance of *Culicoides* species groups [26]. Joint distribution models have been used to analyze community data for different species [76]. At the time of writing this manuscript, there were no reports of the use of joint distributional models for data analysis of *Culicoides* species. Hierarchical joint distribution models in a Bayesian framework are advantageous for analyzing community data compared to distance-based analyses [31]. There will always be inter-species and intraspecies interaction, and this determines the presence/absence or high/low abundance of a species. Therefore, joint distribution models are more reliable than other distance-based metrics (NMDS, PCA, etc.) [31]. Here we propose the use of hierarchical joint distribution models to analyze data on *Culicoides* species to draw inferences about the importance of species interaction and the influence of climatic factors on the abundance of these vectors of important livestock pathogens.

## Figures and Tables

**Table 1:** Site location and number of traps placed, and number of collections made.

S. no	Wildlife sanctuary sites, domestic sites, and at interface	Number of sites for trap placement	Number of collections made
Wildlife sites			
1.	Bannerghatta Biological Park	4	4 sites × 8 months = 32 collections
2.	Rannebennur Black Buck Sanctuary	5	5 sites × 8 months = 40 collections
3.	Biligiri Rangaswamy Temple Tiger Reserve	6	6 sites × 5 months = 30 collections

Domestic sites			
4.	Ramagondanahalli Village in Bengaluru	1	1 site × 8 months = 8 collections

Wildlife—domestic interface			
5.	Gangapur Village near Rannebennur Black Buck Sanctuary	1	1 site × 8 months = 8 collections
6.	HP Colony near Bannerghatta Biological Park	1	1 site × 8 months = 8 collections
	Total	30	129

**Table 2:** Presence and absence of different *Culicoides* species at all the sites.

Site	*C. actoni*	*C. anopheles*	*C. arakawae*	*C. brevitarsis*	*C. boophagus*	*C. circumscriptus*	*C. clavipalpis*	*C. fulvus*	*C. huffi*	*C. imicola*	*C. innoxius*	*C. orientalis*	*C. oxystoma*	*C. palpifer*	*C. palpisimilis*	*C. peregrinus*	*C. similis*	*C. Wadai*	*Total species*
B_1_W	–	+	+	+	–	+	+	+	+	+	–	+	+	+	+	+	–	–	13
B_2_W	–	–	–	+	–	+	–	+	+	+	+	+	+	–	–	–	–	–	08
B_3_W	+	+	+	+	–	+	+	+	+	+	–	–	+	–	+	+	+	+	14
B_4_W	–	+	–	+	–	+	–	+	–	+	–	+	+	–	–	+	–	–	08
B_5_I	+	+	–	+	+	+	–	+	+	+	+	+	+	+	+	+	+	–	14
R_1_W	+	–	+	–	–	+	–	+	+	+	–	–	+	–	+	–	+	–	09
R_2_W	–	–	+	–	–	+	+	–	–	+	–	–	+	–	+	+	+	–	08
R_3_W	+	–	+	–	–	+	+	+	+	+	–	–	+	–	+	–	+	–	10
R_4_W	–	–	+	+	–	+	+	–	–	+	–	–	+	–	+	–	+	–	08
R_5_I	–	+	+	+	–	–	+	–	–	+	+	–	+	–	+	+	+	–	10
T_1_W	+	–	+	+	–	+	+	–	+	+	–	–	+	–	–	+	–	+	10
T_2_W	+	–	+	–	–	+	+	–	+	+	+	+	+	–	–	–	–	–	09
T_3_W	–	–	+	–	–	–	+	–	+	–	+	+	+	–	–	–	–	–	06
T_4_W	–	–	+	–	–	+	+	–	+	–	+	–	–	–	–	+	–	–	06
IV_1_D	+	+	–	+	+	–	–	+	–	+	–	+	+	+	–	+	–	–	10

Site abbreviations: B_1_W, B_2_W, B_3_W, B_4_W, B_5_I: sites withing Bannerghatta Biological Park; R_1_W, R_2_W, R_2_W, R_4_W, R_5_I: sites within Ranebennur Black Buck Sanctuary; T_1_W, T_2_W, T_3_W, T_4_W: sites within Biligiri Rangaswamy Temple Tiger Reserve; IV_1_D: Ramagondanahalli Site.

**Table 3:** Shannon and Simpson diversity index of all the sites. Refer to Table 1 for site abbreviations.

Site	Shannon index	Simpson index
B_1_W	1.84	0.77
B_2_W	1.30	0.61
B_3_W	1.26	0.49
B_4_W	0.86	0.52
B_5_I	1.17	0.60
R_1_W	0.33	0.12
R_2_W	0.57	0.23
R_3_W	0.66	0.28
R_4_W	0.81	0.36
R_5_I	0.72	0.41
T_1_W	1.85	0.79
T_2_W	1.87	0.81
T_3_W	1.71	0.80
T_4_W	0.81	0.35
IV_1_D	0.86	0.48

**Table 4:** Comparison of models with different metrics (DIC, WAIC, AIC, and BIC). Negative binomial model with covariates is the best model with the lowest DIC.

	Conditional DIC	WAIC	AIC at post. median	BIC at post. median
Poisson model without covariates	1139.46	1285.27	1400.37	1645.06
Poisson model with covariates	965.26	938.60	1,406.44	2,428.39
Negative binomial model without covariates	913.95	1,167.69	1,288.44	1,597.91
Negative binomial model with covariates	892.32	1,229.95	1,665.17	2,751.90

## Data Availability

The COI barcode sequences of different *Culicoides* species are submitted to GenBank. The authors do not have permission to share abundance data on *Culicoides*.

## Supplementary Materials

Supplementary Materials The maps of all the sites sampled are shown in Figures S1 and S2: Numerous sequence alignments were performed within every species of *Culicoides* (12 species), and overall among 12 different species of *Culicoides* genus showed varying numbers of Indel in the Cox-1 gene of *Culicoides* species (Table S1). The proportion plot of different *Culicoides* shows that *C. oxystoma* is abundant across sites except for three sites within BRT, followed by *C. imicola* as the most abundant species across the sites. In BRT, *C. innoxius* is more dominant compared to other species. The proportion of other species varies across each site (Figure S3). A hierarchical cluster analysis of the *Culicoides* species shows that *C. imicola* and *C. oxystoma* are in different clusters, and the remaining species are in one cluster (Figure S4). A hierarchical cluster analysis of the sites shows that site 3 of Bannerghatta Biological Park (B_3_W) is in the second cluster and village next to Bannerghatta National Park (B_5_I) and Ramagondanahalli village (IV_1_D) in a cluster. All other sites fall into cluster 1 (Figure S5). The plots of the residual analysis of the Poisson model with no covariates is shown in Figure S6. Wing pattern of voucher specimens obtained after nondestructive DNA extraction is shown in Figure S7.
