# Supplementary material for: Abundance and Diversity of Culicoides Species (Diptera: Ceratopogonidae) in Different Forest Landscapes of Karnataka, India: Implications for Culicoides Borne Diseases
Source: Transbound Emerg Dis. 2023 Aug 31;2023:6250963. doi: 10.1155/2023/6250963 (PMC12017147; doi:10.1155/2023/6250963)
Supplement: Supplementary Materials — The maps of all the sites sampled are shown in Figures S1 and S2: Numerous sequence alignments were performed within every species of Culicoides (12 species), and overall among 12 different species of Culicoides genus showed varying numbers of Indel in the Cox-1 gene of Culicoides species (Table S1). The proportion plot of different Culicoides shows that C. oxystoma is abundant across sites except for three sites within BRT, followed by C. imicola as the most abundant species across the sites. In BRT, C. innoxius is more dominant compared to other species. The proportion of other species varies across each site (Figure S3). A hierarchical cluster analysis of the Culicoides species shows that C. imicola and C. oxystoma are in different clusters, and the remaining species are in one cluster (Figure S4). A hierarchical cluster analysis of the sites shows that site 3 of Bannerghatta Biological Park (B_3_W) is in the second cluster and village next to Bannerghatta National Park (B_5_I) and Ramagondanahalli village (IV_1_D) in a cluster. All other sites fall into cluster 1 (Figure S5). The plots of the residual analysis of the Poisson model with no covariates is shown in Figure S6. Wing pattern of voucher specimens obtained after nondestructive DNA extraction is shown in Figure S7. [file 6250963.f1.docx]

**Supplementary information**

**Abundance and diversity of *Culicoides* species (Diptera: Ceratopogonidae) in different forest landscapes of Karnataka, India: Implications for *Culicoides* borne diseases**

Archana, M^1^., Nayankumar^1^., Raja Manikandan., Arpita G M^1^., Taniya Ghosal^2^, Abhijit Mazumdar^3^., Divakar Hemadri^4^., P.P. Sengupta^4^., Meenaxi Prasad^5^., YN Reddy^6^., YKM Reddy^7^., Janofer Ummer^4^., Jyoti Misri^8^., H. Rahman^9^., B.R. Shome^4^., Sathish B Shivachandra^4^ and Mohammed Mudassar Chanda^4🖂^


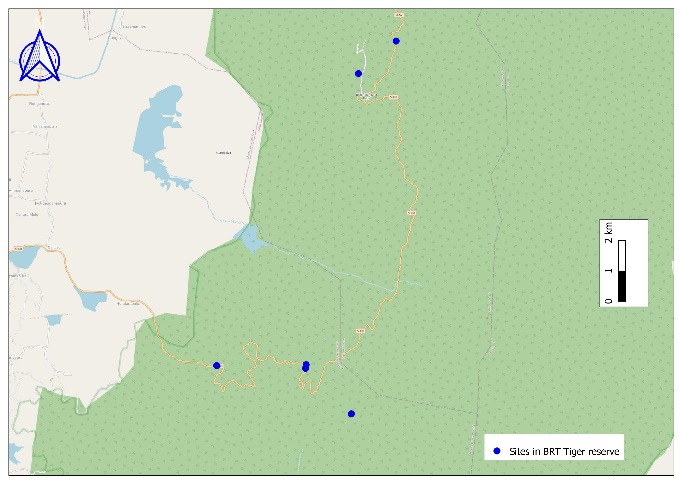

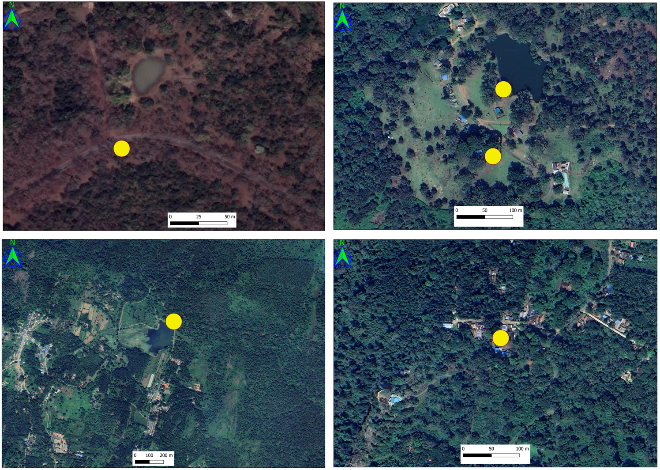


*Supplementary Figure 1: Sites within BRT tiger reserve*


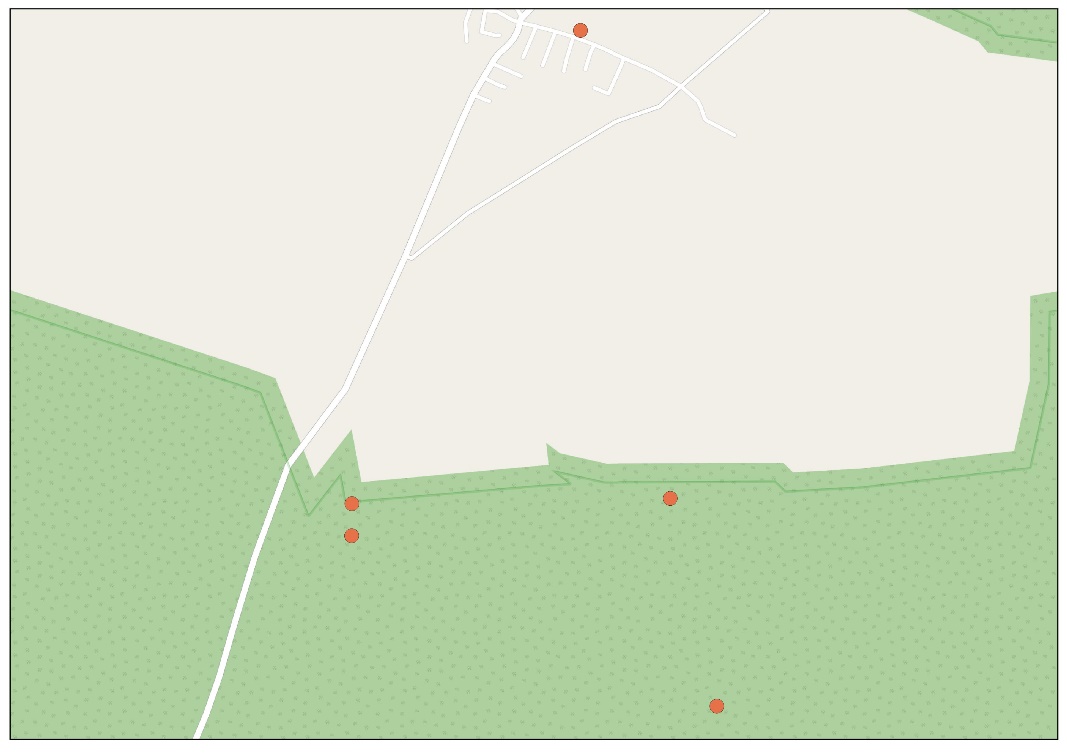


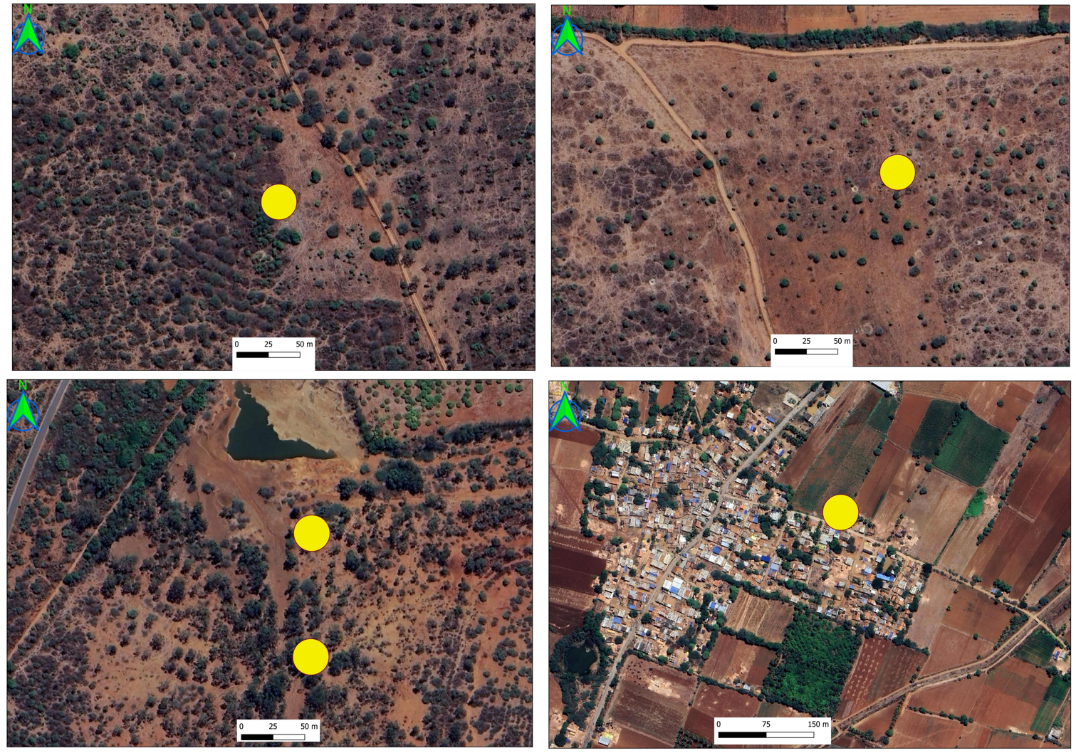


*Supplementary Figure 2: Sites within Rannebennur blackbuck sanctuary*


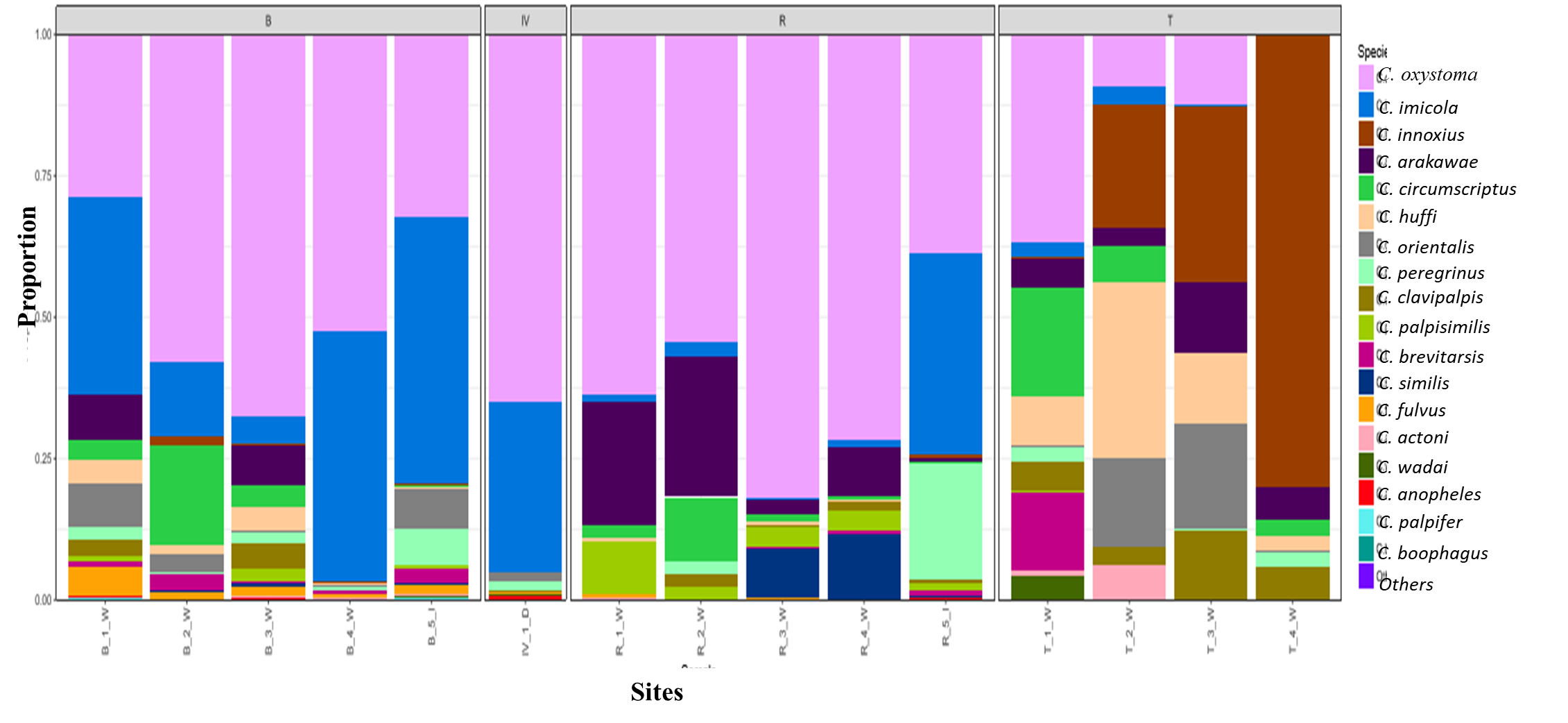


*Supplementary Figure 3: Proportion of different culicoides species at each site. B: sites within Bannerghatta biological park, IV: Ramagondanahalli village (domestic site), R: Rannebennur black buck sanctuary and T: Biligiri Rangaswamy Tiger (BRT) Reserve site*

**Table S1.** Details of the sequences used for the phylogenetic tree construction

| **S. No** | **Culicoides species** | **Cox1 gene sequence available in NCBI** | **Cox1 gene sequence deposited in this study** | **Total number of Cox1 gene sequence used for phylogenetic tree construction** |
| --- | --- | --- | --- | --- |
| 1. | *Culicoides oxystoma* | 9 | 4 | 13 (Tree 1) |
| 2. | *Culicoides actoni* | 16 | - | 16 (Tree 2) |
| 3. | *Culicoides anophelis* | 2 | 2 | 4 |
| 4. | *Culicoides arakawae* | 2 | 2 | 4 |
| 5. | *Culicoides brevitarsis* | 11 | - | 11 (Tree 3) |
| 6. | *Culicoides circumscriptus* | 9 | 3 | 12 (Tree 4) |
| 7. | *Culicoides fluvus* | 10 | 1 | 11 (Tree 5) |
| 8. | *Culicoides huffi* | 6 | 1 | 7 (Tree 6) |
| 8. | *Culicoides imicola* | 24 | 2 | 26 (Tree 7) |
| 9. | *Culicoides innoxius* | 2 | 2 | 4 |
| 10. | *Culicoides orientalis* | 4 | - | 4 |
| 11 | *Culicoides peregrinus* | 6 | 5 | 11 (Tree 8) |
| 12. | *Culicoides similis* | 8 | 3 | 11 (Tree 9) |
|  |  | 109 | 25 | 134 |


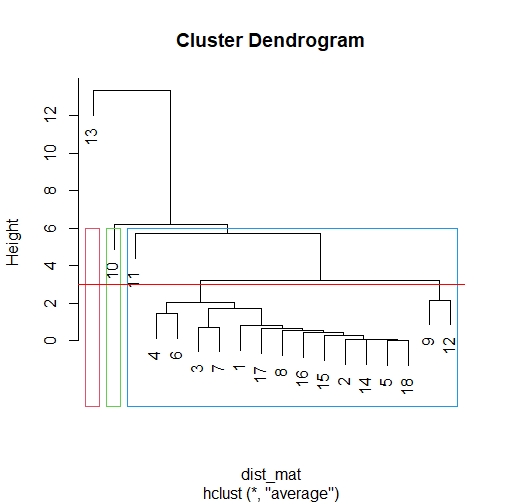


*Supplementary Figure 4: Cluster dendrogram to show clustering of different Culicoides species. Three clusters were choosen.* ***Cluster1:*** *C. actoni (1), C.anopheles (2), C.arakawae (3), C. brevitarsis (4), C.boophagus (5), C.circumscriptus (6), C.clavipalpis (7), C.fulvus (9) C.huffi (11), C.innoxius (12) C. orientalis (14), C.palpifer ( 15), C. peregrinus (16), C.similis (17) C.wadai (18)* ***Cluster 2:*** *C.imicola (10)* ***Cluster 3:*** *C.oxystoma (13)*


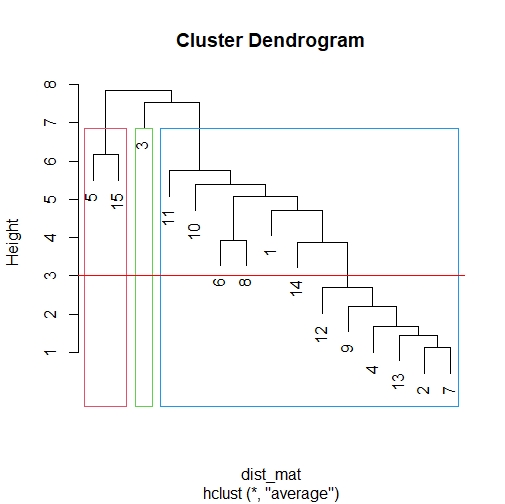


*Supplementary Figure 5: Cluster dendrogram to show clustering of sites with similar habitat. Three clusters were chosen. Cluster 1: B_1_W (1), B_2_W (2), B_4_W (4), R_1_W (6), R_2_W (7), R_3_W (8), R_4_W (9), R_5_I (10), T_1_W (11), T_2_W (12), T_3_W (13), T_4_W (14), Cluster 2: B_3_W , (3)Cluster 3: B_5_I (5), IV_1_D (15). Abbreviations of the sites are given in Figure 3.*


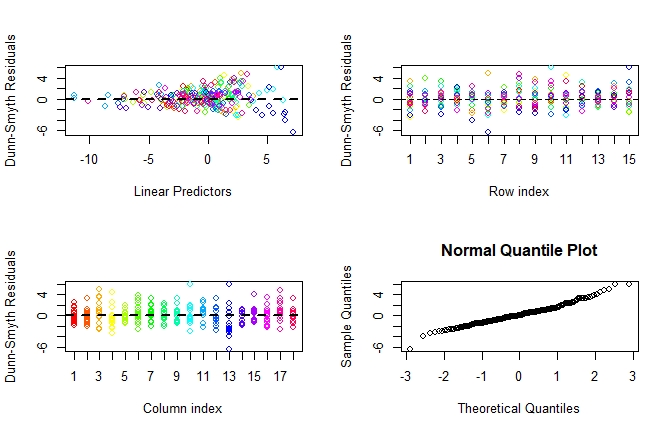


*Supplementary Figure 6: Plots of residual analysis of the Poisson model without covariates. Each color represents different Culicoides species; Top left: Dunn-Smyth residuals vs linear predictors, Top right: Dunn-Smyth residuals vs. row index; Bottom left: Dunn-Smyth residuals vs. column index; Bottom right: Normal quantile plot of Dunn-Smyth residuals. Funneling effect is observed in the top left plot indicating overdispersion.*

*
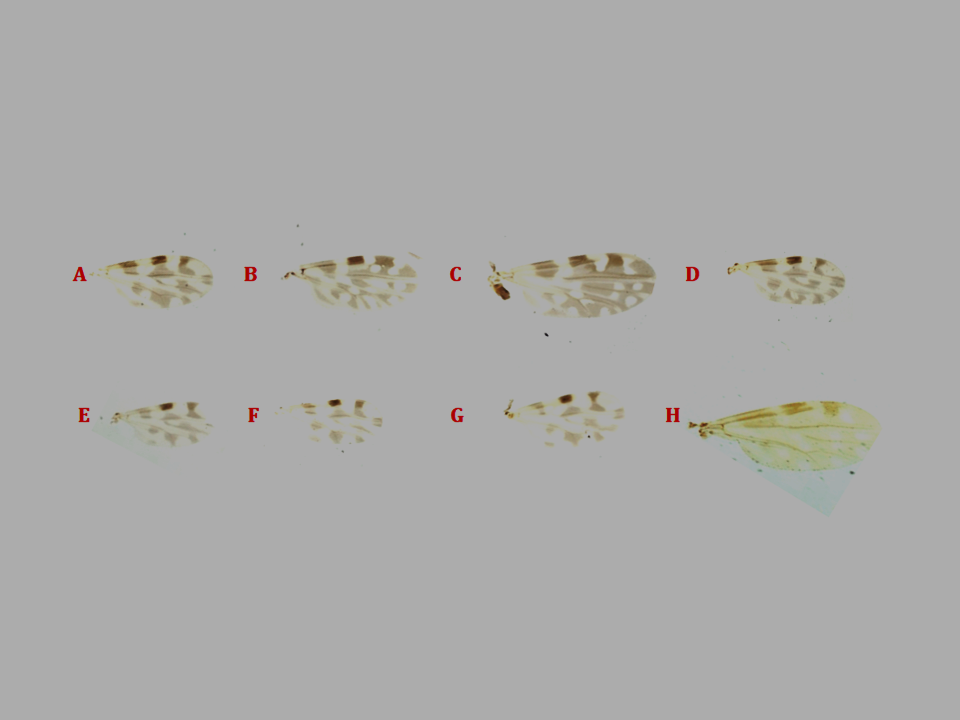
*

*Supplementary Figure 7: Wing pattern of different culicoides species voucher specimens.*

*A: C.orientalis, B: C.Oxystoma, C:C.peregrinus, D: C.boophagus, E: C.brevitarsis, F: C.fulvus, G: C.imicola, H: C.innoxius*
